# Whole brain functional connectivity using phase locking measures of resting state magnetoencephalography

**DOI:** 10.3389/fnins.2014.00141

**Published:** 2014-06-11

**Authors:** Benjamin T. Schmidt, Avniel S. Ghuman, Theodore J. Huppert

**Affiliations:** ^1^Department of Bioengineering, University of PittsburghPittsburgh, PA, USA; ^2^Departments of Neurosurgery and Neurobiology, University of PittsburghPittsburgh, PA, USA; ^3^Department of Radiology, University of PittsburghPittsburgh, PA, USA

**Keywords:** magnetoencephalography, phase locking value, eigenvector centrality, *k*-means clustering, functionally defined regions, functional connectivity, graph theory

## Abstract

The analysis of spontaneous functional connectivity (sFC) reveals the statistical connections between regions of the brain consistent with underlying functional communication networks within the brain. In this work, we describe the implementation of a complete all-to-all network analysis of resting state neuronal activity from magnetoencephalography (MEG). Using graph theory to define networks at the dipole level, we established functionally defined regions by *k*-means clustering cortical surface locations using Eigenvector centrality (EVC) scores from the all-to-all adjacency model. Permutation testing was used to estimate regions with statistically significant connections compared to empty room data, which adjusts for spatial dependencies introduced by the MEG inverse problem. In order to test this model, we performed a series of numerical simulations investigating the effects of the MEG reconstruction on connectivity estimates. We subsequently applied the approach to subject data to investigate the effectiveness of our method in obtaining whole brain networks. Our findings indicated that our model provides statistically robust estimates of functional region networks. Application of our phase locking network methodology to real data produced networks with similar connectivity to previously published findings, specifically, we found connections between contralateral areas of the arcuate fasciculus that have been previously investigated. The use of data-driven methods for neuroscientific investigations provides a new tool for researchers in identifying and characterizing whole brain functional connectivity networks.

## Introduction

Spontaneous functional connectivity (sFC) has become a critical tool for cognitive neuroscience (Lang et al., 2012). sFC is based on the analysis of the statistical relationships between spontaneous temporal fluctuations in brain signals between regions of the brain and has been proposed to reflect underlying neural communications between such regions (Fling et al., 2012; Kinnison et al., 2012). As a tool for neuroscientific research, sFC has provided insight to understand what the brain is doing outside of a task context (Fox and Raichle, 2007; Greicius et al., 2009). The use of sFC has become an important and widely used tool to examine functional connectivity in the typical brain (Fox and Raichle, 2007), to chart neural development (Smyser et al., 2011) and to characterize abnormal brain communication in a host of neurological and psychiatric disorders, such as stroke (Carter et al., 2012), Parkinson's disease (Carbon and Marié, 2003), epilepsy (Wurina et al., 2012), Alzheimer's disease (Li and Wahlund, 2011), autism spectrum disorders (Minshew and Keller, 2010), and is a major part of the Human Connectome Project (Van Essen et al., 2012). Furthermore, studies suggest that abnormalities in spontaneous correlations are associated with neurological dysfunctions and may provide a biological marker for these disorders (Greicius, 2008; Fornito and Bullmore, 2010; Sperling et al., 2010; Carter et al., 2012). Thus, since sFC does not require the patient to perform any explicit task, this approach is of particular clinical interest in populations unable to comply with task instructions such as children, infants, sedated, or comatose patients (Biswal et al., 1995; Arieli et al., 1996; Carbon and Marié, 2003; Sporns et al., 2005; Fox and Raichle, 2007; Boly et al., 2008; Damoiseaux and Greicius, 2009; Smith et al., 2009; De Pasquale et al., 2010; Fornito and Bullmore, 2010; He et al., 2010; Minshew and Keller, 2010; Ghuman et al., 2011, 2013; Smyser et al., 2011; Carter et al., 2012; Hipp et al., 2012; Wurina et al., 2012).

Although spontaneous fluctuations were first observed in blood oxygenation (BOLD) based functional MRI data by Biswal et al. (1995), similar networks have subsequently been observed in blood flow (Zhu et al., 2013), cerebral oxygen metabolism (Lord et al., 2013), and electrophysiological recordings of the brain by both electroencephalography (EEG) (Van Der Kruijs et al., 2012; Engel et al., 2013) and magnetoencephalography (MEG) (Ghuman et al., 2011). Many investigations have utilized functional magnetic resonance imaging (fMRI) due to the high spatial resolution (Biswal et al., 1995; Samuel et al., 1998; Allison et al., 2000; Roebroeck et al., 2011). However, fMRI's low temporal resolution (0.5–2 Hz) and vascularly determined contrast does not lend itself to the direct investigation of high frequency neural cortical activity that have been proposed as a mechanism for information exchange between regions (Gregoriou et al., 2009). In addition, neural timescales are typically much faster than the recording capability of fMRI (Tallon-Baudry et al., 1997).

Magnetoencephalography (MEG) is a non-invasive measure of magnetic fields arising from dendritic activity in neural populations and is typically sampled at greater than 500 Hz allowing for direct investigation of fast neural activity (Hamalainen, 1992). Previous groups have used MEG for the investigation of complex cortical interactions utilizing resting state studies (Ghuman et al., 2011; De Haan et al., 2012b; Westlake et al., 2012). Studies have also demonstrated the efficacy of using phase relationships between cortical areas as a measurement of functional connectivity (Jervis et al., 1983; Tallon-Baudry et al., 1997; Lachaux et al., 1999). Specifically, the phase locking value (PLV) is a measure of the phase synchrony between two time-series, which has been previously applied to resting state connectivity analysis in MEG (Ghuman et al., 2011). As pointed out by Lachaux et al., phase-locking analysis is particularly well-suited for connectivity analysis because it provides a measure of neural signal temporal relationships independent of their signal amplitude (Lachaux et al., 1999). Here we employ PLV as a measure of frequency-specific relationships between cortical regions. This allows for studies that improve our understanding of both the spatial locations of network activity on the cortex as well as allowing us to directly investigate the relationship between frequencies and network communication.

Recently, analysis of sFC has begun to focus on the use of graph metrics to characterization of the spatiotemporal organization of connections and to quantify differences in these networks between patient groups or the brain states within an individual. Previous groups have demonstrated the efficacy of graph theory methods in quantifying these cognitive networks (Strogatz, 2001; Stam et al., 2009; Wang et al., 2012). By employing network statistics, some studies have shown the ability of graph theory metrics to discern abnormalities in populations' network configurations and it has been suggested that dysfunctional cognitive networks may be indicative of wide range of neurocognitive deficits including Alzheimer's disease (Stam et al., 2009; De Haan et al., 2012a; Tijms et al., 2013), drug addiction (Lu and Stein, 2013), schizophrenia (Van Den Heuvel et al., 2010; Siebenhühner et al., 2013), as well as Huntington's disease (Werner et al., 2013). These findings suggest the importance of identification and understanding of aberrant cognitive network functions that may lead to improvements in our understanding of disease processes. These electrophysiological recordings have provided a wealth of information about the cognitive processes that can be unlocked using data-driven approaches to identify important connections.

One of the challenges to analysis of data-driven methods on high-density electrophysiological recordings is the overwhelming dimensionality of the data. Methods have been developed and applied to a wide variety of topics including the recent application to neuroscience datasets that leverage the use of data-mining and graph theory methods upon resting state connectivity data sets and has allowed for a richer data-driven analysis of spontaneous connectivity (Stam et al., 2009; Rubinov and Sporns, 2010; Wang et al., 2012).

In this work, we present a method for quantifying simultaneous whole brain cortical network interactions. To capture multiple neurophysiologically relevant frequency bands, we used MEG because of its high temporal resolution. Following cortical reconstruction, we calculate the PLV between every pair of cortical dipole locations thus producing whole brain functional connectivity networks between every cortical surface location yielding a high-dimensional graph of network activity. We compute functionally defined regions by clustering the results of an eigenvector centrality (EVC) calculation on the all-by-all dipole connectivity from which we apply non-parametric statistical methods to estimate the functional network activity. We show that these whole brain functional connectivity networks can be identified utilizing our method and further, we validate our method using numerical simulations. Finally, we apply our whole brain phase locking network methodology to real data and show that expected network patterns emerge with our data-driven approach.

## Materials and methods

In this section, we will describe the implementation and testing of our method to characterize functional networks from all-to-all analysis of phase-locking connections from MEG data. We will first describe the overall architecture of our analysis and then provide evidence of its validity, characteristics, and limitations from numerical simulations. Finally, we will demonstrate this approach with application to experimentally acquired MEG resting state data.

**Figure 1:**
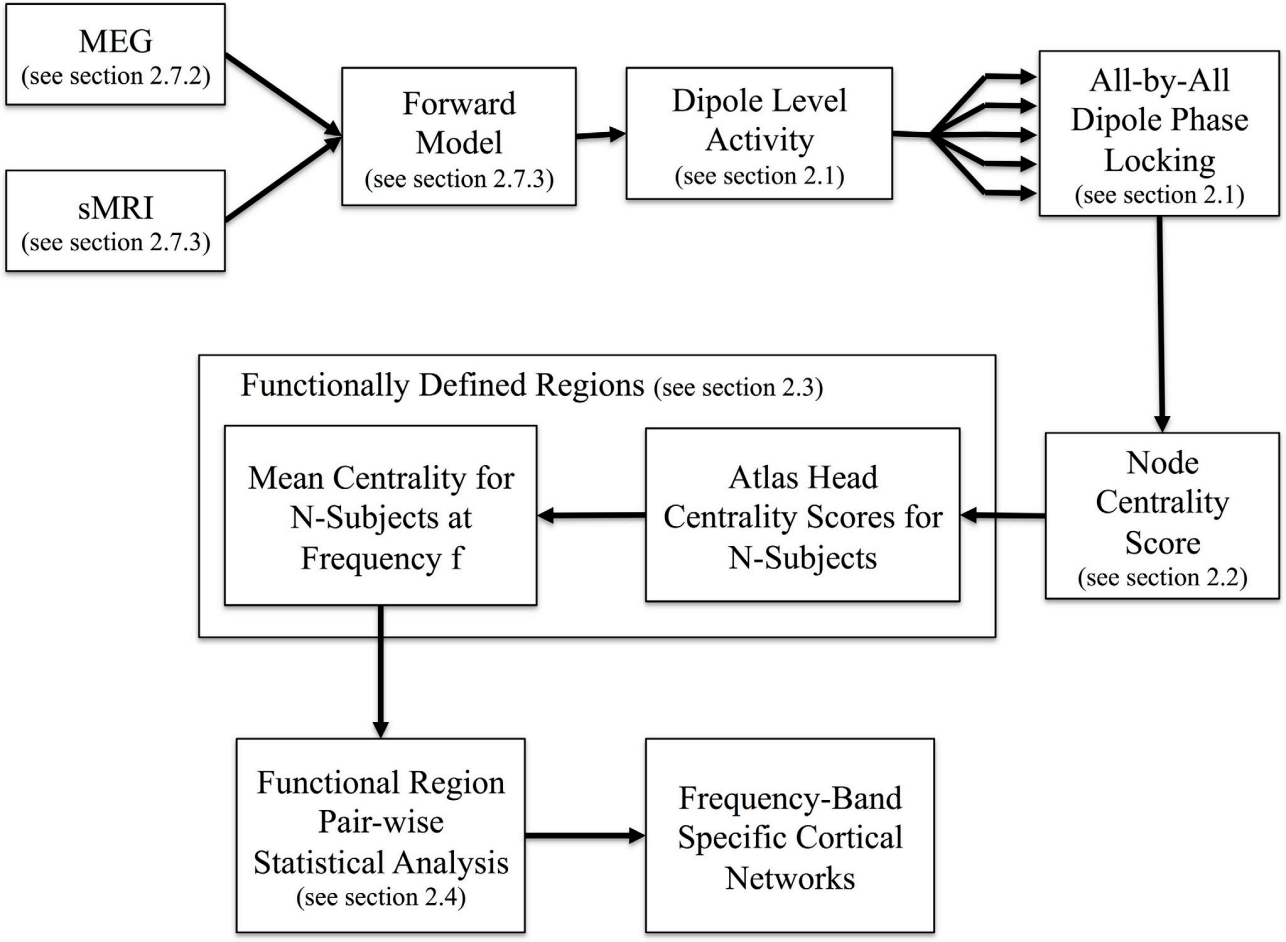
**Overview of method**. Using inputs from MEG recordings and MRI structural information we reconstruct neural activity at each dipole on the cortical surface. The all-by-all dipole level phase locking is then calculated at the given frequency of interest for each subject before registration to a common atlas. Identification of functionally defined regions proceeds by clustering the centrality scores of the phase locking network in the atlas space. Using the regions as input, we then compute the all-by-all pairwise region-level network which yields the frequency band specific cortical networks.

A schematic outline of our proposed analysis stream is presented in Figure 1. In brief, after preprocessing of MEG data to remove external noise, eye-blink, cardiac, and motion artifacts (described in section 2.7.2), the MEG data from the sensors was reconstructed into brain (source) space using a minimum norm solution, yielding a movie of neural time-courses for each location in the reconstructed brain image as described in section 2.7.2. For each pair-wise combination of reconstructed dipole locations in the brain (in our example, 8196 × 8196 dipole locations), a time-averaged PLV was calculated for each temporal frequency of interest (details follow in section Resting State Phase Locking). The resulting all-by-all matrix describes the statistical coupling between all pairs of dipoles in the brain. Based on this matrix, the relative importance of each dipole is quantified using an EVC score which describes the connectedness of each dipole to other brain regions (section Dipole Centrality). EVC is one of several centrality methods and was chosen because it assigns relatively high centrality scores to graph nodes that connect to other important nodes. Nearby dipoles on the surface of the cortex are expected to exhibit similar centrality scores because of both non-independence of the dipole estimates due to the nature of the MEG source reconstruction (inverse) problem and the intrinsic compartmentalized nature of the brain. Therefore, the next step of the analysis was to cluster nearby dipoles with similar scores into functionally defined regions (section Anatomically Biased Functionally Defined Regions). Finally, the statistical probability of connections between regions is analyzed using non-parametric permutation testing to define the statistical graph of connected regions in the brain (section Statistical Testing of Regional Connections). The end result is a description of the functionally defined regions functional connectivity.

The key features of our proposed method include network characterization that leverages the pairwise phase relationships of the entire cortical surface as input, identification of regions determined via clustering of dipoles with similar network interactions amongst the population as well as utilizing a non-parametric statistic to account for the spatial correlations resulting from the MEG reconstruction method. These steps will now be described in more detail in the following sections. To characterize our model, we used both numerically simulated data and experimental data.

### Resting state phase locking

The application of phase locking analysis to MEG has been previously described in Tallon-Baudry et al. (1997), Lachaux et al. (1999), Ghuman et al. (2011). Phase locking is a measure of the propensity for two time series signals to maintain constant phase separation with each other over a period of time. Measures of resting state phase locking between time series electrophysiology recordings are an indication of the temporal variability of the phase differences between those time series at a given frequency. The explicit inclusion of frequency specific information provides insights into networks as a function of frequency.

To calculate the phase locking value, two time series are first spectrally decomposed at a given frequency, *f*_0_, to obtain an instantaneous phase estimate at each time point. Calculation of instantaneous phase was performed using a continuous wavelet transform (Tallon-Baudry et al., 1997). A Morlet wavelet was chosen because it is Gaussian shaped in both the temporal and spectral domains. The Morlet wavelet is described by the following equation:

$$w(t,\;f_{0})={\left(\sigma_{t}\sqrt{\pi }\right)}^{-1/2}e^{-t^{2}/2\sigma_{t}^{2}}e^{2i\pi f_{0}t}$$

The spectral bandwidth, σ_*f*_, is then obtain via σ_*f*_ = 1/(2πσ_*t*_) and a constant ratio enforced via *f*/σ_*f*_ = *c* where we choose *c* = 7 consistent with Tallon-Baudry et al. (1997), because it provides good spectral resolution at lower neurophysiological frequency ranges while still maintaining resolution at higher frequencies. Our target for neurophysiologically relevant frequencies of interest was integer values between 5 and 40 Hz and we choose our value for *c* so that it provides good resolution at these frequencies. Characteristic of wavelets, the temporal and spectral bandwidth is a function of the frequency. As an example, at *f* = 10 Hz the σ_*t*_ = 111 ms and σ_*f*_ = 1.4 Hz and similarly at *f* = 30 Hz then σ_*t*_ = 37 ms and σ_*f*_ = 4.3 Hz showing that the temporal and spectral bandwidth of the wavelet are a function of the center frequency.

After obtaining instantaneous phase estimates the time-averaged PLV can be computed via the following equation:

$$PLV=\frac{1}{N}\left|\sum_{n\;=\;1}^{N}e^{i(\theta_{1}(n)-\theta_{2}(n))}\right|$$

where *N* is the number of sampled time points and θ_1_ and θ_2_ are the instantaneous phase values at time point *n*. Phase locking values range from 0 for a random phase relationship to 1 for a fixed phase relationship. Phase locking is an un-directed measure and is therefore symmetric [i.e., *PLV*(s_1_, s_2_) = *PLV*(s_2_, s_1_)].

Whole brain phase locking networks are computed between each pair of time series measurements on the cortical surface. Due to the symmetry of phase locking, we need only compute one PLV per pair of time series. For *L* total cortical locations we must compute *L*(*L* − 1)/2 phase locking values to obtain the all by all phase locking network.

### Dipole centrality

The whole brain phase locking network are represented using graph theory in which vertices correspond to cortical dipole locations and phase locking values between vertices correspond to weighted edges between those vertices (Bavelas, 1948; Rubinov and Sporns, 2010). The centrality of a graph is a measure of the relative importance of a node and is used to characterize networks. There are several variations of centrality metrics and in this work, we chose to apply EVC because it incorporates the entire graph structure in determining the relative importance of each node in the network. EVC is used to characterize a node in a network according to the amount of connections that node has with other important nodes (Wink et al., 2012; Binnewijzend et al., 2013). Therefore, in phase locking networks, it provides an estimate of the importance of a single dipole within the whole brain network.

The EVC of the network was obtained by computing the first eigenvector after applying the singular value decomposition of the adjacency matrix of the phase locking network (Lohmann et al., 2010), where the adjacency matrix is the *L* x *L* square matrix in which each entry, *a_i,j_* corresponds to the PLV value between the *i*th and *j*th dipole location. This yields a vector of length n in which each entry, *d_i_*, is the relative importance of the *i*th dipole. For two dipoles in the *n*-length centrality vector, *d_i_* and *d_j_* in which the values of the two entries are such that, *d_i_* > *d_j_*, then we say that the *i*th dipole is more important than the *j*th dipole within the network. The diagonal elements of the phase locking adjacency matrix, which correspond to PLV between a time series and itself, are set to zero corresponding to non-self referential edges (i.e., dipoles do not form network connections with themselves).

### Anatomically biased functionally defined regions

Functionally defined regions were calculated by clustering for spatially contiguous cortical surface dipole locations that contained similar patterns of centrality scores. This is in contrast to using an independent anatomical localizer (Milo et al., 2002; Leicht and Newman, 2008; Wang et al., 2009). The identification of functionally defined regions was motivated by the need to identify starting regions from which to initialize statistical methods for identifying region-based clustering. To that end, we used a clustering algorithm that incorporated both the spatial location as Cartesian coordinates of the dipole on the cortex as well as the EVC scores of each of those dipole locations.

This clustering method was chosen such that regions were spatially smooth along the cortical surface to prevent disjoint functional areas. We used the distance along the manifold surface of the cortex to constrain clusters of neighboring points. In this way, dipoles along the same gyrus would be more related than locations spanning across a sulcus, thus better matching our expectation about the compartmentalization of the brain. In order to do this, we used coordinates in a spherically registered representation of the cortex as partial inputs to the clustering algorithm. As described in section 2.7.3, MEG source localization was performed based on anatomical information from MRI data, which was processed and segmented using the FreeSurfer software (Dale et al., 1999; Fischl et al., 1999). The final stages of FreeSurfer analysis involve the extraction, inflation, and registration of the tessellated cortical surface into a spherical space, which forms the basis for dipole locations. We used a cosine distance metric for calculating the distances between vertices so that regions were contiguous by minimizing the angle between them. Cosine distance is given by the following equation:

$$\begin{array}{l} distance\left(A,\;B\right)=\cos\left(\gamma_{A,B}\right)=\frac{A\cdot B}{\left\|A\right\|\left\|B\right\|}\\ \;A=\left[\frac{{\text{x}}_{A}-{\bar{x}}_{A}}{\sigma_{x_{A}}},\frac{{\text{y}}_{A}-{\bar{y}}_{A}}{\sigma_{y_{A}}}\frac{{\text{z}}_{A}-z_{A}}{\sigma_{z_{A}}}\frac{{\text{EVC}}_{A}-{\bar{EVC}}_{A}}{\sigma_{EVC_{A}}}\right]\\ \;B=\left[\frac{{\text{x}}_{B}-{\bar{x}}_{B}}{\sigma_{x_{B}}},\frac{{\text{y}}_{B}-{\bar{y}}_{B}}{\sigma_{y_{B}}}\frac{{\text{z}}_{B}-{\bar{z}}_{B}}{\sigma_{z_{B}}}\frac{{\text{EVC}}_{B}-{\bar{EVC}}_{B}}{\sigma_{EVC_{B}}}\right] \end{array}$$

where γ_*A,B*_ is the angle between the two vectors *A* and *B* that are *n*-dimensional vectors from the *k*-means algorithm and correspond to a four dimensional vector with first three components being the surface coordinates on the brain surface and the last term of the EVC of the location. Each column in *A* and *B* is zero mean and unit variance, with *x*_*A*_ indicating the mean of *x* coordinates in the vector *A*, and σ_*x*_*A*__ is the variance of the *x* coordinate elements of *A*. ||*A*|| indicates the Euclidean norm of the vector *A*.

Using *k*-means clustering allowed us to compute cluster membership of the four-dimensional input space (Macqueen, 1967). *K*-means yields *k* clusters from the original data set and attempts to minimize the equation:

$$arg\;\underset{S}{\min}\sum_{i\;=\;1}^{k}\sum_{x_{j}\in S_{i}}distance(x_{j},\;\mu_{i})$$

where *S* is the configuration of the clusters, *x_j_*, is the location of each original dipole location and corresponding EVC value, μ_*i*_ is the centroid mean at the *i*th cluster, and the distance function assigns a scalar value to the distance between *x_j_* and μ_*i*_. The *x_j_* are zero mean and unit variance prior to starting the estimation procedure. The heuristic attempts to find the best configuration of *S* such that we minimize the errors.

To calculate the *k*-mean clusters we employed an iterative solver from MATLAB (MathWorks Natick, MA) that uses a heuristic algorithm (Macqueen, 1967). The solver works by first assigning *k* random data inputs as centroid locations. The iteration then proceeds in two steps. First, each point is assigned to its closest centroid according to the distance metric. Then, each cluster's centroid is recalculated given the new input values corresponding to that cluster and the closest data point to the new centroid is assigned as the centroid of that cluster. This method continues until convergence is reached in which the centroid assignments are unchanged between iterations or the maximum number of iterations is exceeded. We choose a maximum number of iterations for solution convergence at 1000 iterations.

**Figure 2:**
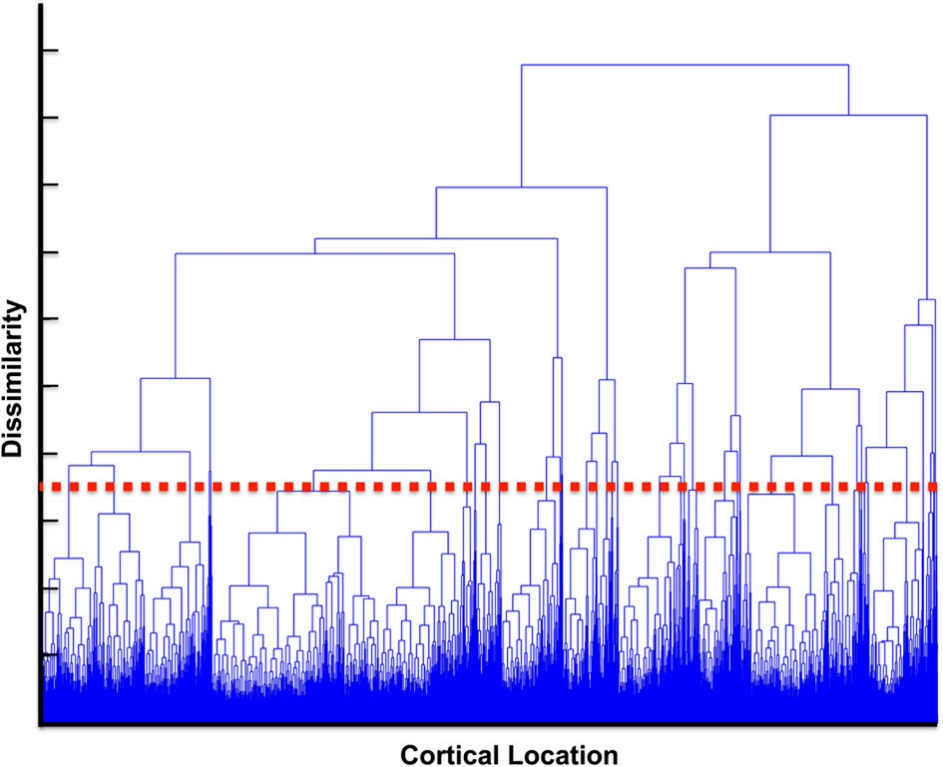
**Dendrogram of cluster similarity**. The dendrogram was computed based upon iteratively aggregating clusters. On the y-axis the dissimilarity between clusters is displayed. For larger clusters (increased y-axis) the dissimilarity increases as clusters contain more diverse values. The red line indicates a choice of *k* for our *k*-means algorithm that provided tractable computationally complexity (increasing the number of clusters) vs. homogeneity of the values in the cluster.

When choosing the number of clusters, *k*, for use in the *k-means* algorithm there is an inherent tradeoff between computational complexity and the homogeneity of clusters. For increasing values of *k*, the number of permutation tests that must be run increases as the square of the number of clusters while the spatial extent of each cluster decreases. This leads to increases in the complexity of the calculation making it more difficult to achieve significance, however smaller clusters would yield greater similarity between dipoles contained in the cluster and provide more granular detail of functional regions. To ascertain an appropriate *k*-value, we calculated a dendrogram in which we iteratively generated clusters using successively smaller *k*-values upon the obtained centrality scores (Caliński, 2005). Briefly, at each iteration the number of clusters is reduced by one and correspondingly two clusters are aggregated into a combined cluster with a lower similarity score (as a result of inhomogeneity of the precursor clusters). We can characterize this level of dissimilarity by computing 1-similarity between the merged clusters. The choice of *k* = 40 corresponded to cutoff in which we chose the number of clusters small enough to be computationally tractable while still providing neuroscientifically relevant network structures such that clusters were small enough to make interpretation possible. This value was used for both the real and simulated data. The dendrogram is shown is Figure 2, where increasing y-axis results in greater dissimilarity between clusters being joined at lower levels. The red dashed line in the figure indicates our choice for the number of clusters.

### Statistical testing of regional connections

The final step of our approach was to perform statistical tests on the connections between functionally defined regions. We determined significant connections between these functionally defined regions via a non-parametric permutation test. Each pairwise grouping of functional regions was compared across all subjects in the resting state scans to the empty room scans. To calculate statistically significant connections between pairs of regions we used a non-parametric permutation that explicitly tests the underlying distribution of dipole level phase locking values between the two functionally defined regions.

The permutation test follows the method described in Maris et al. (Maris and Oostenveld, 2007). For *S* subjects, the permutation test is as follows for a given pair of regions. First, the phase locking values are obtained for those PLV values that connect the two regions (i.e., edges that connect the two regions). This population is aggregated over the subjects to obtain a distribution of phase locking values by binning PLV values to create a histogram. We obtained this distribution for both the resting state and empty room scans. The null hypothesis in our permutation test is that the empty room scan data and the subject recording are the same. In permutation tests, any metric may be chosen and here we used the difference in means between the two populations as our test statistic because of its low computational overhead (Maris and Oostenveld, 2007). Our observed test statistic is therefore the difference in the mean between the two populations of empty room and resting state data.

Next, we performed permutation of the data in the two populations, empty room and subject data, and applying our test statistic to every permutation of the combined data. From this permutation calculation we obtained a distribution of test statistics. At a given critical value, α, we found the test statistic that corresponded to that critical value. Finally, we tested if our observed test statistic is more extreme than our critical value. If the observed test is greater than the critical value we rejected the null hypothesis that the two groups come from the same distribution. This indicates a statistically significant connection between the two functional regions and therefore a positive connection between the two clusters.

For *k* functionally defined regions obtained via our clustering method, there are *k*(*k* − 1)/2 pairs of regions on which to perform this permutation test. In addition, due to the large number of dipole connections between two regions, we used a Monte Carlo (MC) sampling of the permutation test to reduce the number of permutations required by iteratively sampling the permutation space until successive iterations yielded a small change in outcome the distribution of test statistics. For our stopping criteria, we required a minimum of 5000 permutations after which we stopped the MC algorithm when there was less than a 0.01% change in the distribution. This method has been shown to asymptotically equivalent to the full permutation test (Dwass, 1957; Bickel and van Zwet, 1987). Following computation of all pairwise connections at the functional region level, we obtain a graph network of connections between functional regions at the given frequency.

### Description of numerical simulations

In order to explore the performance and sensitivity of our approach, we first generated a set of synthetic simulation MEG data. The purpose of these simulations was to quantify the sensitivity and specificity of the proposed methodology. In particular, the estimation of source space signals in MEG introduces a spatial blurring and subsequent point spread function due to the MEG reconstruction procedure. This effect has been shown to introduce false-positive correlations between nearby cortical surface points (Ghuman et al., 2011). In order to evaluate the rate of false positives generated by the spatial point spread, we conducted simulations at nine dipole pair locations to examine our method's ability to recover the original signal locations. We performed numerical simulations in which dipole signals were generated and projected into MEG sensor space. We applied our proposed method to test the differences between the simulated dipole information and the networks produces via our method.

Simulations of source space signals were conducted utilizing the MNE software package using a method similar to Ghuman et al. (2011). Briefly, simulations were conducted by generating 10 Hz sinusoids at two dipole locations with the same frequency at each location that, following the addition of noise, are able to provide a realistic simulation of subject data. One dipole location was fixed on the cortical surface within the parietal lobe and for each of the nine simulation distances the location of the second dipole was moved incrementally closer starting in the frontal lobe so that we could evaluate the effect of inter-dipole distance on our method. Inter-dipole distances ranged from a 10 to 1 cm. In sensor space, noise was added according to a covariance matrix obtained from empty room recordings. Five minutes of data were generated for each simulation at 250 Hz with 306 channels in MEG sensor space.

The sinusoidal amplitude was determined by matching the PLV values obtained in real data. First, we calculated the average PLV in subject data at the distances used in our simulation. Next we set the amplitude of the sinusoidal generators such that the PLV at the equivalent distances had the same PLV following our simulation procedure. Following the addition of noise and the reconstruction onto the cortical surface, the original sinusoids were contaminated with realistic noise in sensor space data. The sinusoidal amplitudes we used ranged from 1.5 × 10^−8^ at the largest distance to 3.5 × 10^−7^ at the shortest distance. This has the effect of fixing the SNR of the simulation to be comparable to PLV present in real data by matching the distribution of PLV signals (this corresponded to a simulation SNR of −26 dB). A total of nine positions were used in simulations. For each dipole position pair, 10 repetitions were performed.

Due to regularization of the MEG source localization problem, the resulting images will be spatially blurred. In order to account for the point-spread function of the inverse problem in the estimate of the false-positive rate of the model, sensitivity and specificity analysis was performed by two alternative definitions of positive clusters in which two clusters were statistically connected. First, as those clusters that contained the original dipole and second as a more relaxed criteria of clusters which were located within a specific distance of the dipole of interest (i.e., the dipole used as a sinusoidal generator) based on the full-width half maximum (FWHM) extent of the reconstructed dipole in empty room data.

**Figure 3:**
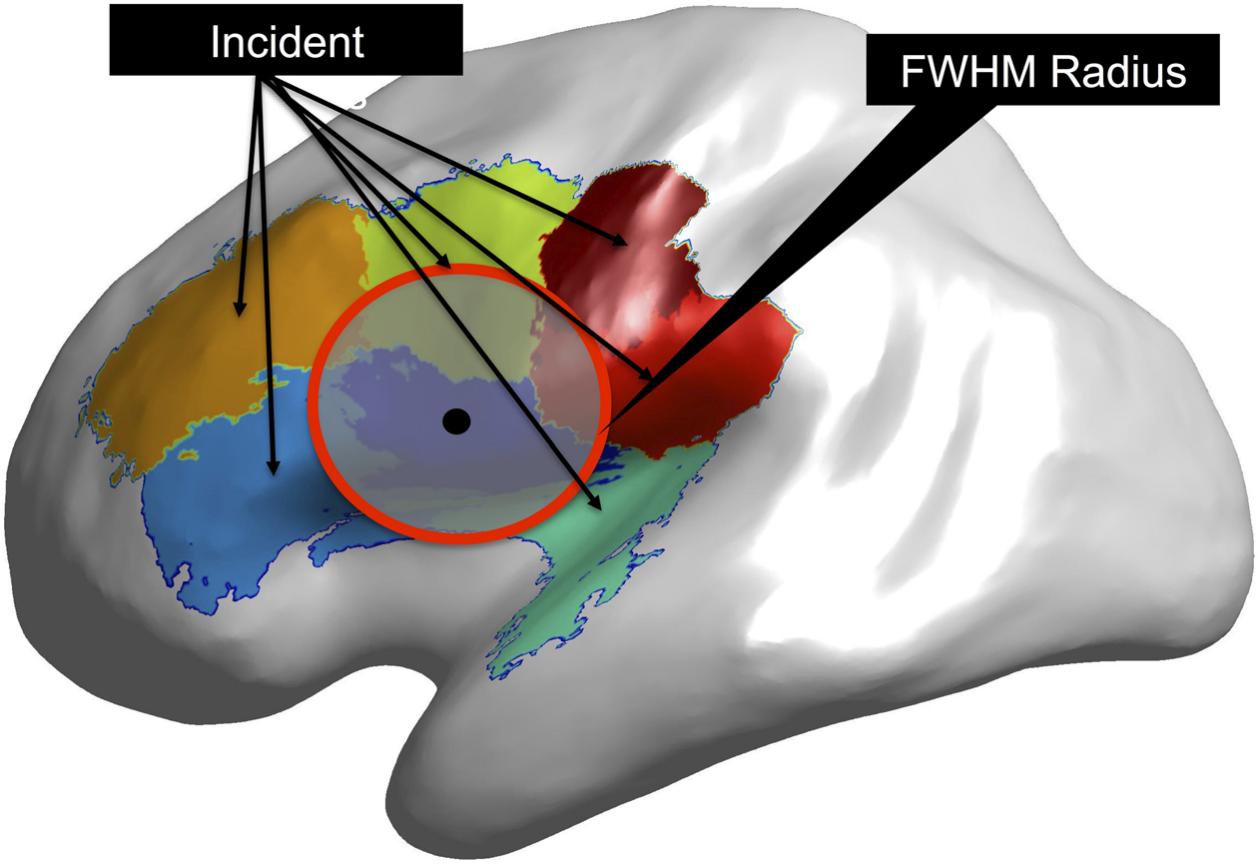
**Calculation of Full Width Half Max (FWHM)**. For each dipole used in the simulation (black dot) the FWHM is calculated (red circle) as list of points on the cortical surface within that radius. Clusters that are incident within the FWHM radius are part of the FWHM true positive rate.

For each dipole location we calculated the full width half maximum (FWHM) radius around the given dipole in Cartesian space (see Figure 3). We began by calculating the distribution of inter-dipole phase locking values from the dipole of interest to every other dipole and ordering the distribution by distance between each dipole pair. One distribution was obtained for each of the nine dipole positions used in the simulation. We then calculated the FWHM for each position of the distributions by determining the inter-dipole distance that corresponded to half of the maximal inter-dipole PLV value.

For each cluster, we computed whether any of the dipoles that were members of that cluster fell within the FWHM distance from the simulation point. Clusters that possessed this property were considered to be within the FWHM radius of the around the dipole location. A representation of this process is shown in Results where the source dipole location is shown and an accompanying overlay of the clusters with surfaces that contain areas that are within the FWHM radius. This provided an additional metric for understanding the role of reduced resolution resulting from the point spread function. Namely, we are able to test the resolution of the original signal generating dipole as a function of distance from that dipole.

### Evaluation of PLV characteristics

In order to characterize the robustness of our method, we characterized the effects of signal modulations on our ability to resolve significant connections between network clusters. We simulated a 306 × 306 two-dimensional grid of locations each with an individual time series. Each time series in the grid included iid Gaussian noise with the same SNR from real subject data. At two fixed 10 × 10 grid locations we generated a sinusoidal signal, similar to our above simulation, to simulate brain activity in those areas. In this reduced model, we applied our methodology to identify clusters of activity and generate significant phase locking networks. To understand the effect of different signal modulations on our ability to resolve connections, we included four perturbation conditions of the two simulated brain activity regions. Using this model we tested how different signal manipulations affect the network graphs.

The four manipulations consisted of SNR variations, signal modulation, phase variances, and frequency offsets. First we modified the signal strength of the sinusoid by modifying the amplitude of the sinusoid to adjust the SNR relative to the real data (2.0 × SNR_real_, 1.0×, 0.5×, 0.1× amplitude multiples of amplitude used in the above simulation). To test signal modulations we included a low-frequency carrier signal and tested at multiple low-frequency modulators (0.1, 0.5, 1, 5 Hz). We also created phase variances and frequency offset simulations. The signals in the two regions were generated according to the following equations:

$$\begin{array}{l} s_{1}\left(t\right)=\sin\text{​}\left({\text{w}}_{1}\text{t}+\Phi_{1}\right)+\varepsilon \\ s_{2}\left(t\right)=\sin\text{​}\left({\text{w}}_{2}\text{t}+\Phi_{2}\right)+\varepsilon \end{array}$$

where *s*_1_ and *s*_2_, are used to generate signals in the active regions, *w*, is the frequency of the underlying sinusoid and ε is iid Gaussian noise. Phase variances were investigated by setting *w*_1_ = *w*_2_, and allowing Φ to be a uniform random distribution with mean zero. That is, phases randomly drawn from a uniform distribution on the range −*M* to *M* (values of *M*: π/10, π/4, π/2, and π). Finally, to understand our methods' ability to differentiate different frequencies, we set *w*_1_ ≠ *w*_2_ while fixing the phase variance with Φ = 0. We set *w*_1_ = 25 Hz and varied *w*_2_ at 20, 15, 10, 1 Hz. Each of the perturbation settings were repeated 50 times producing a total of 800 simulations (4 conditions × 4 settings × 50 simulations). The results of the simulations were analyzed using a receiver operating characteristic (ROC) of the true positive vs. false-positive significant cluster connections. We defined a true positive to be a significant connection between the two clusters containing the active regions regardless of the perturbation condition. This definition allowed us to compare the methodologies resolution across conditions and perturbations.

### Experimental methods

#### Subject population

Resting state (spontaneous) MEG signals were recorded from five healthy human subjects (3 male, ages 25–45 and 2 female, ages 20–25). All subjects were right-handed. Each subject participated in two 5 min resting state MEG scans which were concatenated together in source space prior to pre-processing. In each scan the subject was instructed to fixate on a centrally placed cross-hair, eyes open while sitting upright. Each scan lasted 5 min. Prior to subject scanning an empty room dataset was collected which consisted of 10 min of MEG data collection without a subject present in the MEG. The institutional review board of the University of Pittsburgh approved all study procedures. Written consent was obtained from each participant prior to participation in this study.

#### MEG recording and preprocessing

A 306-channel Elekta Neuromag scanner was used for recording of neuromagnetic signals. Head position coils were placed on the scalp to determine head position during recording.

Temporal spatial signal separation (tSSS) was employed to remove external magnetic field contributions in the signal recordings which can lead to spurious signals which appear to have originated from physiological changes but are in fact artifacts external to the MEG sensors (Taulu and Simola, 2006). Time series MEG recordings were preprocessed to remove excess noise and artifacts prior to connectivity analysis. The MEG signals were first bandpass filtered using cutoffs of 1 and 50 Hz to remove the 60 Hz line noise and slow drifts. MEG scans were sampled at 1000 Hz and subsequently downsampled to 250 Hz.

#### Structural MRI and MEG source localization

Structural MRI images were obtained for each subject using a 3-Tesla whole body MRI scanner (Siemens). A T1-weighted brain volume was recorded for each subject. Following coregistration between MEG fiducial markers and the MRI structural images a cortical surface model was obtained using the FreeSurfer™ software package. A topologically accurate cortical surface mesh consisting of approximately 150,000 vertices per hemisphere were subsequently downsampled to 4096 vertices per hemisphere for use in inverse reconstruction.

Reconstruction of cortical dipole activity from MEG sensor measurements is an ill posed problem but an estimation procedure has been described in (Hämäläinen et al., 1993). Briefly, we can estimate this reconstruction by utilizing the minimum norm estimator using the MNE software package. Our data model is given by:

$$\hat{y}(t)=Wx(t)$$

where *x*(*t*) is a time series of MEG sensor space recordings and $\hat{y}(t)$ is a time series of dipole location estimations. The minimum *L*_2_ norm solution for *W* is then given by:

$$W={\text{RA}}^{T}{\left(ARA^{T}+\lambda^{2}C\right)}^{-1}$$

where *A* is the gain matrix of the forward problem and λ is a regularization parameter and is given by λ^2^ = 1/*SNR*. *C* represents the covariance of the noise in the sensors and *R* is the covariance matrix of the sources (Ghuman et al., 2011; Gramfort et al., 2014). The depth-dependent decay of MEG signals was accounted for by including a depth factor of 0.8 onto the *R* matrix (Lin et al., 2006). Furthermore, due to the typical orientation of cortical neurons to be normal to the surface an orientation constraint was applied to *R* such that those components that are tangential to the surface were multiplied by 0.4 and those normal to surface were multiplied by 1 and finally only the normal component is taken. Following the procedure from Ghuman et al., we calculated the noise covariance matrices from an empty room scan of MEG which was recorded absent a subject in order to maintain spontaneous neural activity following cortical reconstruction (Ghuman et al., 2011).

Subject recordings were registered and resampled onto a common template that resulted in a registered cortical space. The all-by-all phase-locking adjacency matrix was then calculated from each subject's source space data using 8196 dipoles. Each calculation took approximately 2 h per 5-min data file (16.7 million PLV computations). Phase locking adjacency matrices were calculated for each subject and then averaged in the common atlas space to produce a single centrality value at a given frequency prior to clustering.

## Results

### Numerical simulations

Here we present our results of the numerical simulation studies. We first show the networks identified via our method with respect to the simulated network configuration. Next we look at the effects of the point-spread function on our ability to resolve networks at successively smaller distances between the two simulated point sources.

**Figure 4:**
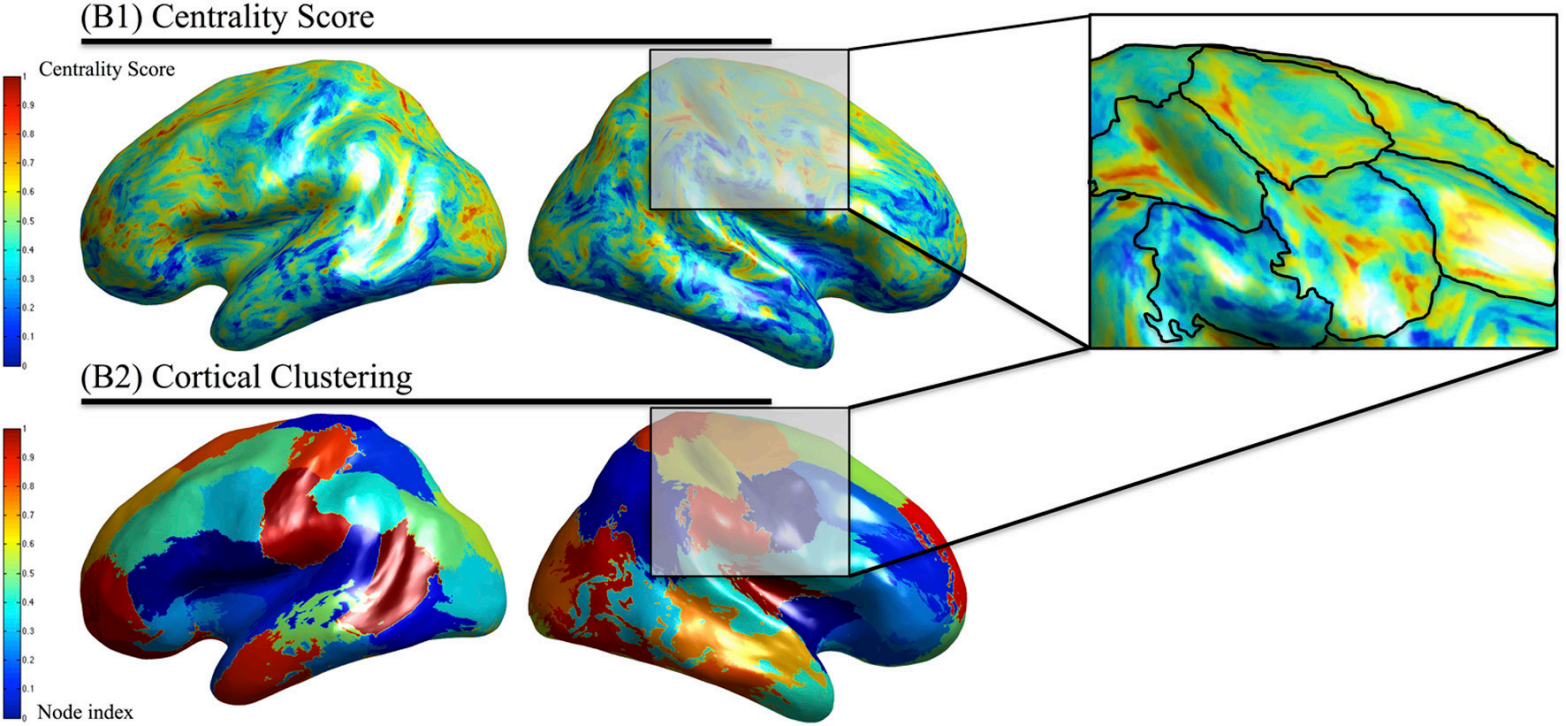
**Identification of functionally defined regions in subject data**. The eigenvector centrality at each cortical location averaged across multiple subjects (above) is used as input to the clustering algorithm that identified regions with similar centrality scores that are nearby on the cortical surface (below). The overlaid regions are depicted on the centrality scores (right).

Functionally defined regions are determined according to the membership of dipole locations to a specific cluster. Each region is spatially contiguous along the cortical surface and possesses dipoles that have similar centrality scores. In the top part of Figure 4, the centrality score is displayed on the cortical surface in which higher values (red) indicate a greater importance of that dipole within the phase locking graph. The lower portion of Figure 4 shows the result of clustering on the centrality scores to determine the functionally defined regions where surface colors are used to represent difference clusters. Finally, Figure 4 shows an overlay of the centrality scores with the clustering method showing similar centrality-value surface dipoles within the functionally defined regions.

#### Performance of data-driven methods

**Figure 5:**
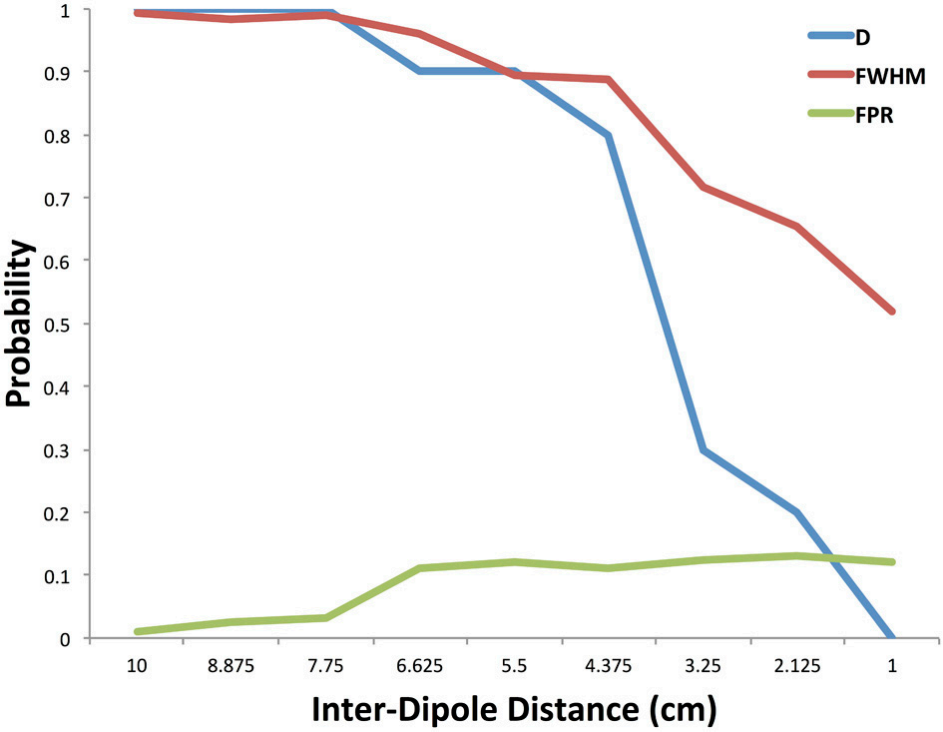
**Method performance in simulation**. The probability of the cluster containing the dipole being significantly connected is plotted with the probability that at least one cluster within the FWHM was significantly connected. These are plotted as a function of the distance between the two original dipoles used in generating the simulation. In addition, the false positive rate is shown for clusters significantly connecting outside the FWHM.

The results of our numerical simulations are shown in Figure 5. The figure shows the probability of the cluster that contains the first simulated dipole point source to be connected to the accompanying cluster at the second simulated dipole point source as a function of the distance between the two dipoles. This plot is the true positive rate (TPR) of the dipole-containing clustering. The figure also presents the probability that at least one of the FWHM clusters is positively connected to another FWHM cluster at a α = 0.05 level, computed using a cumulative distribution function. At large separation distances both estimation methods are able to identify the connection between the two point sources ranging from a 10 cm to 7.75 cm inter-dipole distance. At inter-dipole distances less than 7.75 cm, there is a divergence between the sensitivity measurements such that at 2.25 cm inter-dipole distance the probability of at least one cluster in the FWHM containing a positive connection is 0.65 while the probability that the cluster containing the original dipole is 0.2. The FWHM maintains a higher sensitivity measurement as the inter-dipole distance decreases because of the decreased spatial sensitivity of the FWHM.

Also in Figure 5 we show the false positive rate (FPR) for the combination of the dipole-containing cluster as well as the FWHM clusters. False positives were calculated from those clusters within the FWHM as well as the cluster containing the dipole and similarly for the true negative where all clusters outside the FWHM should be unconnected. There is an increase in the FPR as the inter-dipole distance decreases.

**Figure 6:**
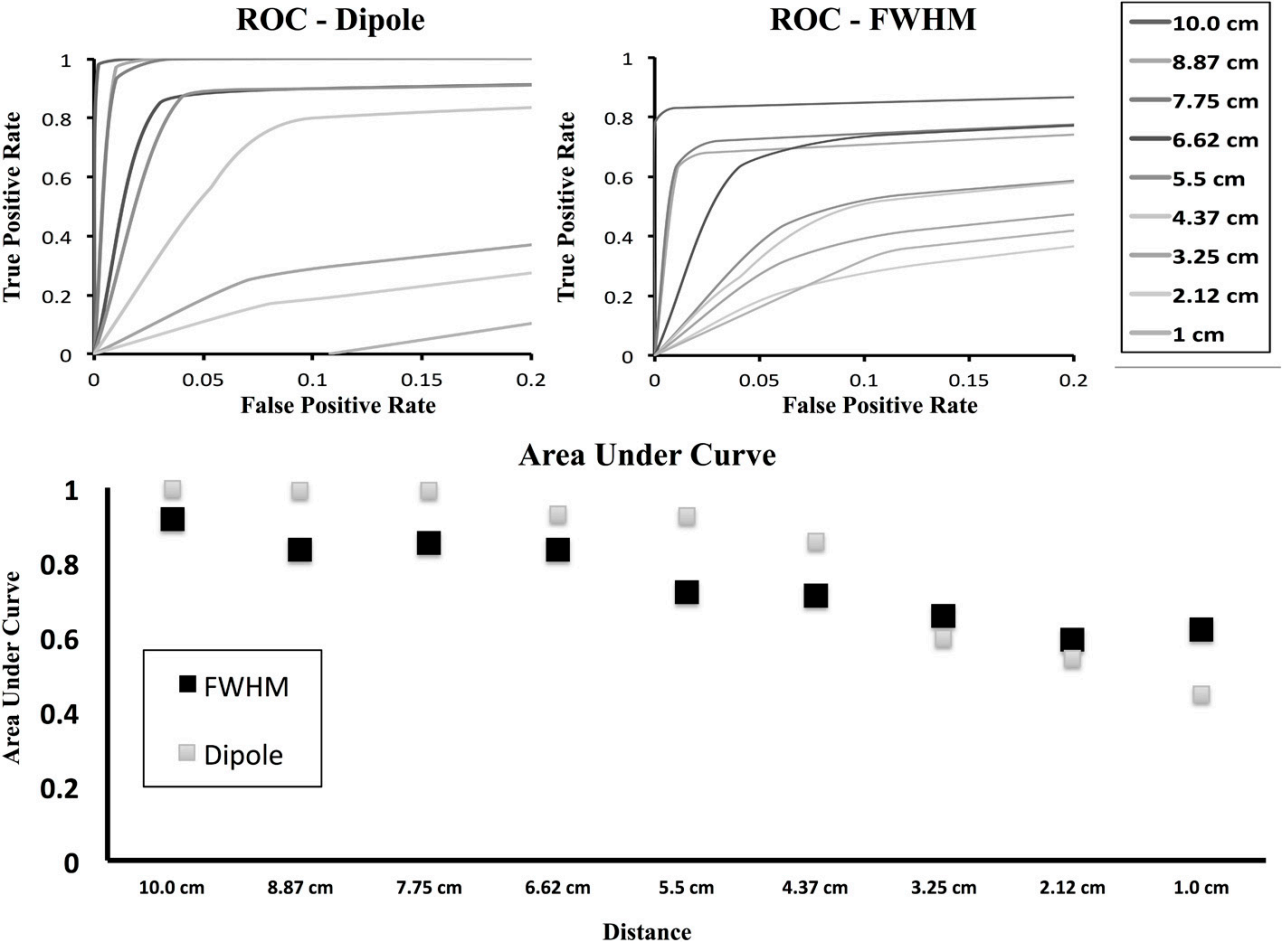
**ROC curves for brain simulation with area under curve**. ROC curves are shown for decreasing inter-dipole distance in the brain simulations for both the cluster containing the originally activated dipole as well as the FWHM region of clusters. As the distance decreases, the number of true positives falls more quickly with only the dipole, but the FWHM maintains resolution at smaller distances.

In Figure 6 we show the ROC curve for both the dipole only condition as well as the FWHM condition. In the dipole condition, as the two generators move closer, the ability of the method to resolve the connection drops as indicated in Figure 5, but the ROC curve shows the rate at which this is occurring. For the FWHM, the ROC curve is moving downwards, but less quickly than the dipole-only.

#### Characterization of errors in cluster spatial location

**Figure 7:**
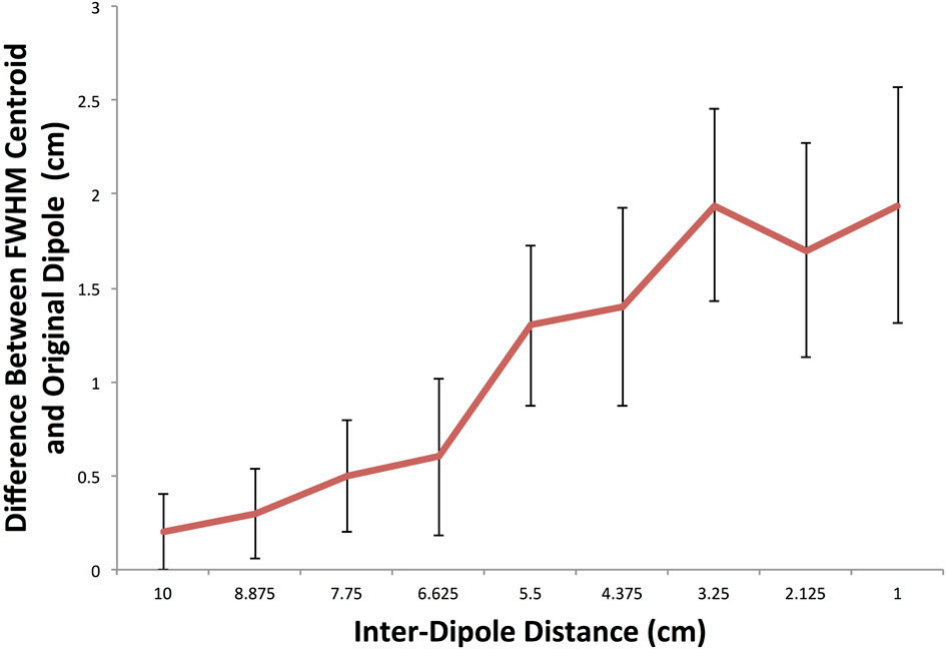
**FWHM centroid location in simulation as a function of inter-dipole distance**. A plot of the difference between the Euclidean distance between the FWHM centroid location and the originally simulated dipole. As the distance between the dipoles decreases, the FWHM clusters tend to be located non-uniformly around the original dipole location.

In order to characterize errors introduced at decreasing inter-dipole distances we calculated the offset between the simulated dipole point source and the center of mass of all FWHM cluster centroids that contained at least one significant connection. The result of this analysis is shown in Figure 7. At the largest inter-dipole distance of 10 cm, there is an average 0.2 cm (standard deviation: 0.21 cm) discrepancy between the centroid locations indicating a good agreement between the centroid of the FWHM clusters and the dipole location indicating a more uniform grouping of significant clusters surrounding the original dipole location. At decreasing distances, there is a divergence in the two centroids as significant FWHM clusters are grouped more distally from the other dipole resulting in a larger difference between the center of mass of the FWHM clusters and the dipole location. This effect is most pronounced at the smallest inter-dipole distance of 1 cm in which the centroid discrepancy is 1.94 cm (standard deviation 0.63 cm).

In agreement with Ghuman et al. (Ghuman et al., 2011), these simulations show that when the distance between two points decreases, the ability to resolve the two points independently decreases. This can be seen as a direct result of the decreased resolution induced by the inverse solution. However, the inclusion of the FWHM radii and the clusters contained therein allows for an increased resolution at smaller inter-dipole distances as seen in Figure 5 as a shift to the right compared to the cluster containing the dipole.

#### PLV characteristics

Our previous simulation used a single sinusoidal generator to which additional noise was added to provide realistic SNR and PLV values based upon realistic data. However, to better understand how variations in the underlying signal affect the outcome of our method, we used a variety of perturbations on the original simulation methodology that included SNR modifications, low frequency signal modulations, phase variances as a function of time as well as our ability to resolve off-frequency sinusoids.

**Figure 8:**
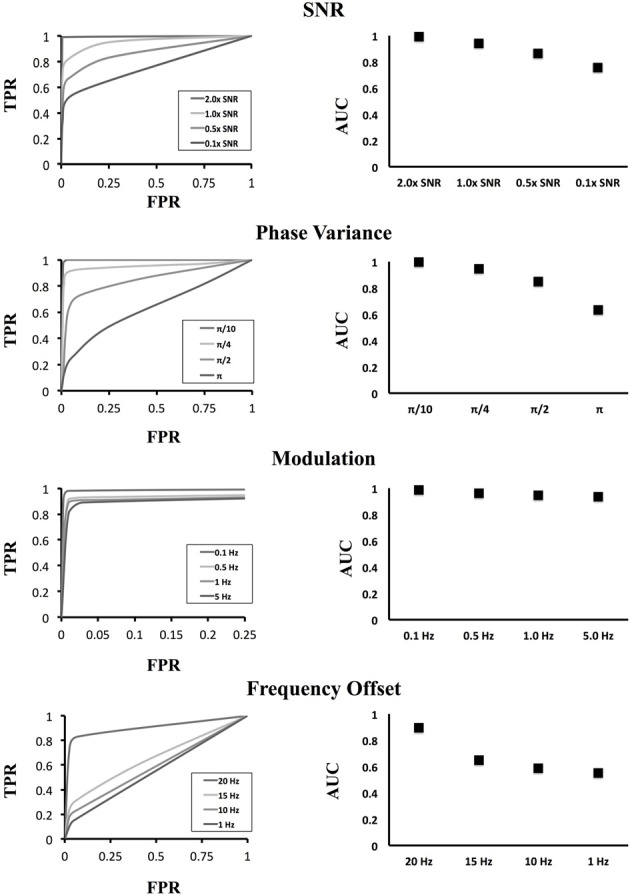
**ROC curves for various signal modulations**. The left column shows the ROC curve for different settings of the simulation while the right column shows the area under the curve at those settings. In the top row, the resolution ability of our method falls as the SNR of the sinusoidal generator decreases. In the second row, we varied the instantaneous phase of the simulated sinusoid with the addition of noise from a uniform distribution. Increasing instantaneous phase variations caused the original sinusoid to degrade resulting in a loss of significant cluster resolution. In the third row, low frequency modulations were added to the signals with very little effect upon the outcomes owing to the time-frequency methods employed. In the last row, sinusoids in the two activated areas were generated using different sinusoidal frequencies. One area was fixed with a frequency of 25 Hz. At the nearby frequency of 20 Hz, we can distinguish connections between the regions; however, as the two frequencies diverged, the wavelet envelope degrades and we can no longer recover significant connections.

**Figure 9:**
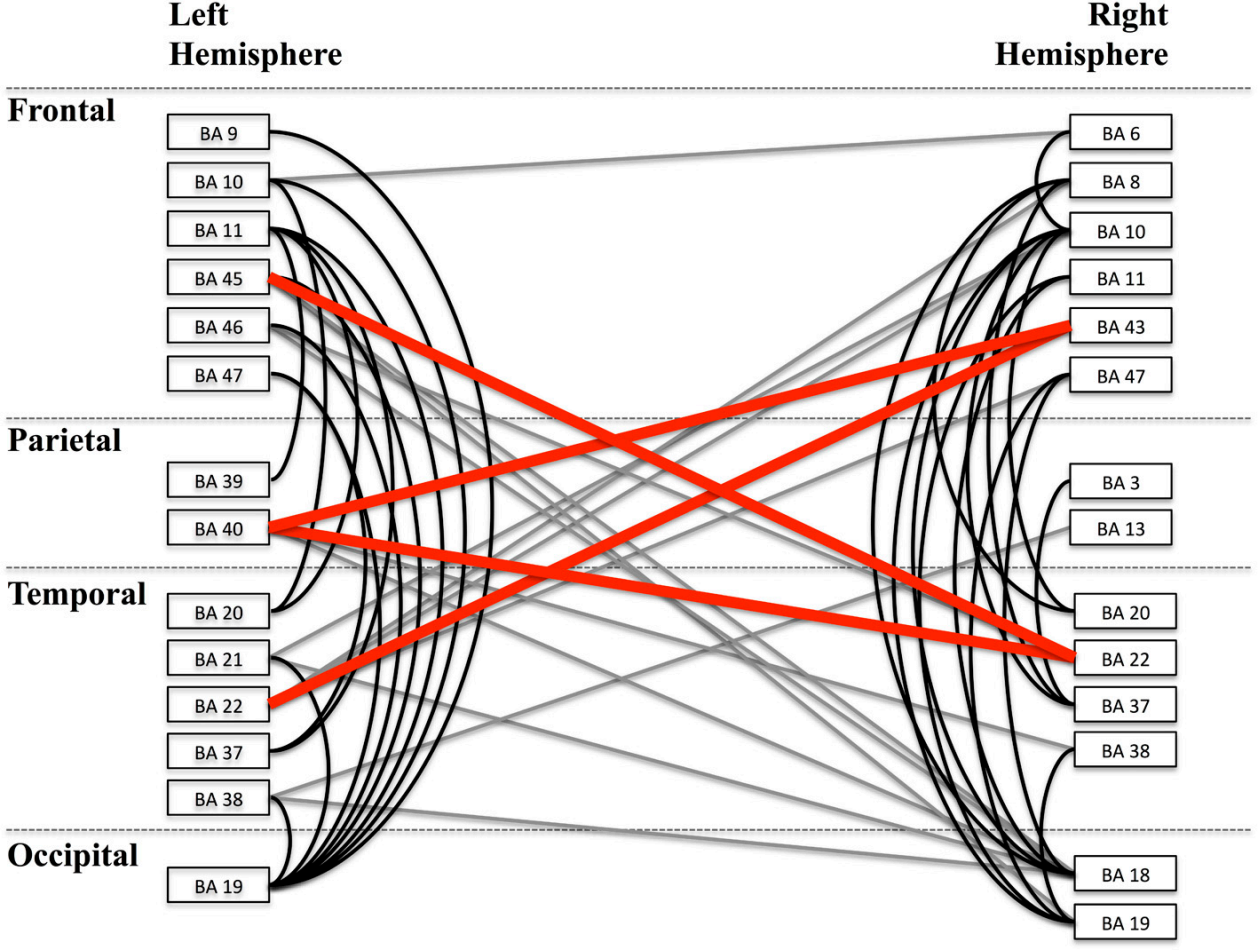
**Alpha band network**. A depiction of the full alpha-band network is shown with each cluster labeled according to the nearest Brodmann area (BA) for visualization. Clusters are organized by lobe. Lines between regions indicate a significant connection between those two regions. Black lines are intra-hemisphere connections while gray lines are inter-hemisphere connections. Red lines depict those connections that are shown in greater detail in Figure 10.

After generating our grid of simulation data (Figure 8), we applied our clustering methodology to identify significant clusters of activity. We considered the 10 × 10 grid that contains the generators (the simulated brain activity) to be a true positive if it connected to the cluster containing the other generator. The results of our simulation are shown in Figure 9. The first curve displays modifications to the amplitude of the sinusoid relative to the background noise and therefore the SNR as a multiple of the real data SNR from above. As the SNR increased, the TPR increased due to the higher resolution, as expected, with decaying SNR the ability of our method to accurately identify the correct clusters subsequently decreases. However, for low-frequency modulations, the ability of our method to identify significant clusters was not affected by the addition of a low-frequency modulator. Because of our use of the wavelet methods for identifying instantaneous frequencies, the addition of a low-frequency modulator on the original signal does not have much of an effect on our outcomes.

We also manipulated the phase variance by explicitly using an instantaneous phase component of the original sinusoid that contains a distribution with mean zero and uniformly distributed about a given band. This has a pronounced effect on graph outcomes as we can see from the ROC curve. This is a direct result of both instantaneous phases changing, and therefore making phase locking more difficult, but it is also a result of changing frequency components. As the instantaneous phase increases in volatility, the ability to uniquely resolve the original frequency becomes more difficult. Finally we investigated unequal frequencies. At a small off-frequency (20 Hz compared to the reference 25 Hz) we are still able to resolve the correct cluster connection as a result of the wavelet time-frequency resolution. That is, small frequency offsets cannot be uniquely distinguished as a result of the characteristics of time-frequency resolution. However, at large frequency offsets the identification of positive clusters drops precipitously. This is again owing to the wavelet method which only resonates over a given frequency band (with a Gaussian envelope) and as a result, off-frequencies do not produce significant connections.

#### Point spread function limitation

One reason for a discrepancy in centroids locations arises because as the inter-dipole distance decreases, the FWHM radius around each point source begins to have overlapping clusters. That is, both dipoles' FWHM can contain the same cluster. As a result, our statistical test will not find a difference between a population and itself and therefore these clusters are precluded from being significantly connected. As a result of this overlap, the significantly connected clusters will tend to group around the distal portions of the FWHM radii of the two dipoles. We see the effect of this distal grouping in Figure 7 as a large discrepancy in the center of mass of the FWHM positive centroids.

The MEG reconstruction method limits the ability to resolve small inter-dipole point sources. However, our numerical simulations show that we are able to obtain good estimates of the reconstructed cortical activity despite this poor resolution. We have also shown that our method produces a small FPR. Finally, the FPR is low through a large number of inter-dipole distances that further validates our statistical methodology and improves this methods utility in neuroscientific investigations. Given our numerical simulation results, we next applied our method to experimental data.

#### Evaluation of statistical methods

We evaluated our statistical method by verifying the estimated critical value after calculating the false discovery rate (FDR). Using two empty room datasets as input, we applied our method to calculate the phase locking network using one dataset as the null hypothesis. We used our chosen critical value of α = 0.05 and subsequently calculated the number of positive connections and repeated this evaluation for 10 pairs of empty room data. From this process we estimated the FDR for our method to have an average FDR of 0.043 with a standard deviation of 0.011.

### Experimental results

All data were visually inspected prior to processing for anomalies. Following preprocessing of the sensor space data, the transformed data were again visually inspected to ensure no abnormalities arose during preprocessing. The network was analyzed at the alpha (8–13 Hz) frequency band to demonstrate the efficacy of our method by averaging PLV activity estimated at integer values within our range.

The alpha band phase locking network is shown in Figure 9. Labeled regions correspond to clusters that were significantly connected to at least one other region. Lines between regions indicate significant phase locking between those regions. The nearest Brodmann area to each functionally defined region was used for labeling. The graph presented is a depiction of the alpha band frequency phase locking connections.

**Figure 10:**
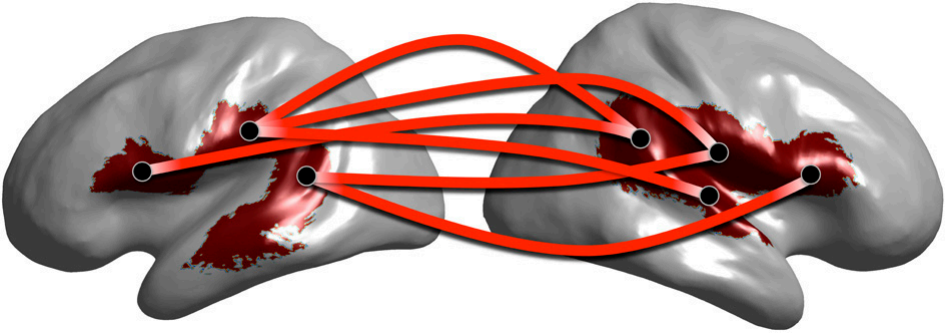
**Graph of alpha band network clusters over the arcuate fasciculus**. Subset of full alpha band network depicting cluster centroids (black dots) overlaid on cluster locations on the cortical surface (red surface). Red lines between centroid locations indicate significant network connections.

In Figure 10 we show a subset of the full graph structure showing clusters overlapping with the arcuate fasciculus. Cortical regions corresponding to the significant clusters are depicted on the cortical surface. Connections are shown between clusters centroids as lines between circular discs that are displayed at the centroid of each region. Our method reveals phase locked connections between contralateral regions of the arcuate fasciculus. Previous studies have shown the arcuate fasciculus plays a role in language (Catani et al., 2005) and furthermore, that functional connections exist between these cortical locations and specifically at the alpha band (Ghuman et al., 2011).

## Discussion

Whole brain functional connectivity on healthy normal subjects has revealed complex network interactions at multiple physiological frequency bands. Limitations arising from the inverse solution that leads to degradation in resolution, and results in a decreased ability to detect spatially nearby functionally connected regions is common in other studies of MEG functional connectivity (Ghuman et al., 2011). To mitigate these effects and provide a tool that neuroscientists can utilize to identify complex cortical network interactions we have introduced a method that incorporates dipole level centrality metrics obtained from graph theory to identify regions with similar connectivity patterns. From these regions we were then able to identify significant connections utilizing a statistical permutation test.

Clustering of graph vertex centrality scores is a novel method for identifying functionally defined regions on the cortical surface. Our choice of spherical clustering methods produces regions that are spatially contiguous without disjointed surfaces and are therefore more easily interpretable. Functionally defined regions have been used previously in establishing cortical locations with overlapping functional behavior (Milo et al., 2002; Cohen et al., 2008; Wang et al., 2009). In general, these methods provide a data-driven method for assessing membership of individual cortical locations and assigning groups to regions. In contrast, anatomically defined regions that are defined based upon the physical connectivity of neural populations (Sporns et al., 2005). The size of the dipole level adjacency matrix necessitated the choice of a centrality measure that is computable in reasonable time upon a roughly 8000 square matrix. Specifically, we used EVC as our measure of similarity between vertex locations as it is a widely used metric within graph theory and it was computationally tractable given the dimensionality of our adjacency matrix.

Our use of permutation tests on the region-by-region statistical analysis provided a test that explicitly accounts for the underlying distributions of phase locking values between regions. This is an important property of permutation tests—that they are non-parametric and do not rely upon the underlying distribution of the data conforming to any specific parametric distribution—and is the primary reason we choose to use the permutation test in our analysis. This is especially important when testing the highly dependent dipole level activity that results from cross talk arising from the MEG reconstruction method. Parametric models of dipole level activity are susceptible to deviations in the expected type I error as a result of violations in the assumptions in the underlying distribution. Importantly, we demonstrated that our method has a low FDR in the simulations producing a small false positive population.

Application of whole brain phase locking estimates to subject data at the alpha band revealed complex network interactions across many areas of the cortical surface. Importantly, these networks arise without a hypothesis driven expectation of the cortical spatial location of the neurophysiological activity. Furthermore, networks that have been studied in literature are revealed by our whole brain analysis utilizing only these data-driven methods. As such, these results provide powerful evidence for the data-driven establishment of alpha band networks. Connections that were previously unknown provide a rich area of future studies to identify their important integration into whole-brain cortical activity.

### Limitations

Owing to the ill-posed nature of the MEG inverse problem, there is no unique solution to the reconstruction of cortical activity. Here we have used the MNE estimation because it makes few assumptions about the covariance structure of the data. MNE is a Tikhonov regularization that uses a scalar identity matrix for the covariance structure. In the context of resting state functional connectivity, the covariance structure is important to understanding the temporal relationships between cortical locations. Alternative methods could provide additional information from resting state data owing to their use of different underlying assumptions. In addition, alternative methods may be more robust to the point spread function. Decreasing the influence of the point spread function on nearby points would allow for more granular assessments of the phase locking between regions. As our simulation results indicated, the point spread function prevents short-distance connections from being significantly detected.

In the present work we have used *k*-means clustering because it is fast, computationally simple, and provides robust estimates for clusters based upon an input space. Given our data-driven focus, we wished to provide a clustering method that establishes membership of every cortical location to a given cluster. In this way, we were able to compute the whole brain functional connectivity mapping. *K*-means is known to produce clusters with similar spatial extents, which can be seen in Figure 4 owing to its variance-based clustering heuristic. This is especially useful in top-down modeling in which we first assign all points to the cluster and eliminate those clusters that are not significant. In addition, *k*-means does not provide a robust mechanism for estimating the number of clusters. In the present work we used a dendrogram to identify a reasonable tradeoff between computational complexity (increasing clusters increases the computational time by the square of the number of clusters) and similarity between dipoles contained within the cluster. Owing to this tradeoff, we have underestimated the total number of clusters. As a result, regions that contained populations of more than one similarity may produce a higher type II error rate.

However, using a bottom-up approach and more complex clustering model one could aggregate related functional locations into significant clusters. Gaussian mixture models have been used in developing these bottom-up models of functionally related cortical locations (Daunizeau and Friston, 2007; Olier et al., 2013). In that work, mesostate models aggregate related dipole-level activity into regions of similar activity and provide a bottom-up model generation technique for understanding functional connectivity. Interestingly, mesostate models can incorporate priors into their analysis, and one such prior could be the outcome of our methodology providing the top-down view of the whole-brain network architecture. In addition, these alternative methods for clustering could provide methods to empirically estimate the number of clusters and eliminating the need for our dendrogram method for estimating a *k* for *k*-means.

We used a time-averaged PLV that aggregates each time-series into a single value. It is well-known that MEG contains many non-stationarities in the data and that those non-stationarities can be directly investigated using windowing methods (Ghuman et al., 2013). Time averaging over the scan period can only capture processes that are jointly stationary. The use of wavelets in our work results in a methodological framework that is not as susceptible to processes that are non-stationary when estimating the instantaneous phase of each signal independently as compared with traditional Fourier methods. However, PLV is still susceptible to signals that are not jointly stationary (i.e., if each signal changes the underlying properties together we are still able to resolve that relationship even if they are each non-stationary processes). Time windowing would provide a method to investigate this property directly by analyzing smaller windows of time (i.e., over a short duration window the signals are jointly stationary).

### Future directions

In this paper we have presented a methodological framework for assessing whole brain functional connectivity networks. Our method is unique in that it attempts to provide simultaneous assessments of the underlying cortical network architecture at specified frequency band without requiring knowledge of the expected network connections. This data-driven approach to whole brain functional connectivity provides a model-free view of cortical networks and provides a starting point for future investigations of individual network connections. We believe that our method is a powerful tool for identifying previously undiscovered networks. However, our method provides a very high-level understanding of whole-brain networks. The combination of generative models of dipole-level activity and our top-down modeling approach would provide researcher the methodological framework to investigate functional connectivity patterns over a large range of scales.

The use of graph theory for analysis of cortical networks provides a toolset for assessing various aspects of brain health and quantitative metrics for characterizing those networks. Future studies could incorporate these graph theory quantification metrics upon revealed networks. For instance, they could be used to assess differences between frequency bands or subject populations. These comparisons would then be capable of providing neuroscientific insights into cognitive processes in a quantitative manner that could ultimately lead to an improved understanding of cognitive disease models.

### Conflict of interest statement

The authors declare that the research was conducted in the absence of any commercial or financial relationships that could be construed as a potential conflict of interest.
